# Injuries and /or trauma due to sexual gender-based violence among survivors in sub-Saharan Africa: a systematic scoping review of research evidence

**DOI:** 10.1186/s13690-024-01307-3

**Published:** 2024-05-21

**Authors:** Desmond Kuupiel, Monsurat A. Lateef, Patience Adzordor, Gugu G. Mchunu, Julian D. Pillay

**Affiliations:** 1 Faculty of Health Sciences, Durban University of Technology, Ritson Campus, Durban, 4001 South Africa; 2 Discipline of Public Health Medicine, School of Nursing and Public Health, University of KwaZulu-Natal, Durban, 4001 South Africa; 3 The John Wesley School of Leadership, Carolina University, 420 S. Broad Street, Winston- Salem, NC 27101 USA; 4 UNiTED Projects, Kpando, Ghana

**Keywords:** Injuries, Trauma, Sexual violence, Gender-based violence, Survivors, Sub-Saharan Africa, Scoping review

## Abstract

**Background:**

Sexual and gender-based violence (SGBV) is a prevalent issue in sub-Saharan Africa (SSA), causing injuries and trauma with severe consequences for survivors. This scoping review aimed to explore the range of research evidence on injuries and trauma resulting from SGBV among survivors in SSA and identify research gaps.

**Methods:**

The review employed the Arksey and O’Malley methodological framework, conducting extensive literature searches across multiple electronic databases using keywords, Boolean operators, medical subject heading terms and manual searches of reference lists. It included studies focusing on injuries and trauma from SGBV, regardless of gender or age, published between 2012 and 2023, and involved an SSA countries. Two authors independently screened articles, performed data extraction and quality appraisal, with discrepancies resolved through discussions or a third author. Descriptive analysis and narrative synthesis were used to report the findings.

**Results:**

After screening 569 potentially eligible articles, 20 studies were included for data extraction and analysis. Of the 20 included studies, most were cross-sectional studies (*n* = 15; 75%) from South Africa (*n* = 11; 55%), and involved women (*n* = 15; 75%). The included studies reported significant burden of injuries and trauma resulting from SGBV, affecting various populations, including sexually abused children, married women, visually impaired women, refugees, and female students. Factors associated with injuries and trauma included the duration of abuse, severity of injuries sustained, marital status, family dynamics, and timing of incidents. SGBV had a significant impact on mental health, leading to post-traumatic stress disorder, depression, anxiety, suicidal ideations, and psychological trauma. Survivors faced challenges in accessing healthcare and support services, particularly in rural areas, with traditional healers sometimes providing the only mental health care available. Disparities were observed between urban and rural areas in the prevalence and patterns of SGBV, with rural women experiencing more repeated sexual assaults and non-genital injuries.

**Conclusion:**

This scoping review highlights the need for targeted interventions to address SGBV and its consequences, improve access to healthcare and support services, and enhance mental health support for survivors. Further research is required to fill existing gaps and develop evidence-based strategies to mitigate the impact of SGBV on survivors in SSA.

**Supplementary Information:**

The online version contains supplementary material available at 10.1186/s13690-024-01307-3.

**Table:** 

**Text box 1. Contributions to the literature**	
This study provides a comprehensive overview of the impact of sexual and gender-based violence on sub-Saharan African survivors, highlighting diverse injuries and trauma across demographics. Identifying research gaps, it informs a research agenda to address knowledge shortcomings. Highlighting the profound mental health impact, the study reveals consequences like post-traumatic stress disorder, depression, anxiety, and suicidal ideations. Additionally, it unveils challenges survivors face in rural healthcare access, offering valuable insights for policymakers and practitioners. Overall, the study enhances understanding of SGBV’s multifaceted toll and calls for targeted interventions and further research in the field.	

## Background

The World Health Organization (WHO) reports that injuries are a growing global public health problem [1]. In 2021, unintentional and violence-related injuries were estimated to cause over 4 million deaths worldwide, accounting for nearly 8% of all deaths [1]. Additionally, injuries are responsible for approximately 10% of all years lived with disability each year [1]. While injuries can result from various causes such as road traffic accidents, falls, drowning, burns, poisoning, and acts of violence, including sexual and gender-based violence (SGBV) [1, 2], SGBV remains a neglected cause of injuries that silently affects the lives of many, especially women [3, 4]. Injuries due to SGBV refer to physical harm or trauma resulting from acts of violence perpetrated based on an individual’s sex or gender. These injuries can encompass a range of physical harm, including but not limited to bruises, cuts, fractures, internal injuries, and sexual trauma (psychological or emotional) [5].

Sexual and gender-based violence is a pervasive issue [6–10] with alarming rates globally, particularly in the WHO Africa and South-East Asia regions with 33% each compared to 20% in the Western Pacific, 22% in high-income countries and Europe, and 31% in the WHO Eastern Mediterranean region [8]. However, this statistic includes only physical and/or sexual violence by an intimate partner alone and does not include other forms of violence [8]. Sexual and gender-based violence encompasses various acts such as sexual assault, rape, intimate partner violence, and harmful traditional practices, all of which have severe physical and psychological consequences for women [9, 11, 12]. The sub-Saharan Africa region has witnessed numerous cases of SGBV perpetrated against vulnerable populations, such as women, children, refugees, and individuals with disabilities, with devastating impacts on their well-being and overall quality of life [13–32].

Understanding the extent and nature of injuries and trauma resulting from SGBV among survivors is crucial in formulating effective interventions, policies, and support systems. Research evidence plays a fundamental role in shaping responses to this pressing public health concern, guiding the development of targeted interventions and preventive measures. However, the available research on injuries and trauma related to SGBV in sub-Saharan Africa remains scattered and diverse, necessitating a comprehensive and systematic review to consolidate and analyse existing knowledge.

A scoping review study would support a valuable research approach to systematically map and describe the existing evidence on injuries and trauma related to SGBV against women in sub-Saharan Africa. In so doing, the scoping review would provide a broader overview of the literature to identify knowledge gaps, key concepts, and various study designs employed in the field, and inform more specific research questions that can be unpacked by way of a systematic review and /or meta-analysis quantitative studies or meta-synthesis of qualitative studies [33, 34]. To our knowledge, current literature shows no evidence of any previous scoping review that has focused on injuries and trauma due SGBV. This study, therefore, conducted a systematic scoping review to explore the scope of research evidence regarding injuries and trauma stemming from SGBV among survivors in sub-Saharan Africa. This research sheds light on the prevalence, patterns, and factors associated with injuries and trauma resulting from SGBV in the region and their impact on survivors.

## Methods

To achieve the objective of this scoping review, we utilised the Arksey and O’Malley methodological framework [35] as a guiding framework for mapping and examining the literature on injuries and trauma associated with SGBV in the context of sub-Saharan Africa. This framework comprises several key steps, including identifying the research question, identifying relevant studies, study selection, data charting and collation, and summarizing and reporting the results [33, 34].

### Identifying the research question

The primary research question guiding this scoping review is as follows: What is the scope of research evidence regarding injuries and trauma resulting from sexual and gender-based violence among survivors in sub-Saharan Africa in the last decade? To ensure the appropriateness and relevance of this question, we employed the Population, Concept, and Context (PCC) framework [36] as part of the study eligibility criteria, which is detailed in Table 1. To comprehensively address the research objective, the scoping review explored the following sub-questions:

**Table 1:** Study eligibility criteria

Eligibility criteria	Inclusion criteria	Exclusion criteria
Population	Individuals of all ages who are victims or survivors of sexual gender-based violence, missing and between sexual and gender-based violence.	
Concept	Injuries due to SGBV: Physical harm or trauma resulting from acts of violence perpetrated based on an individual’s sex or gender. These injuries can encompass a range of physical harm, including but not limited to bruises, cuts, fractures, internal injuries, and sexual trauma (psychological or emotional); as well as their consequences.	Injuries linked to unintentional injuries such as road traffic accidents and disasters and intentional injuries such as Attempted suicide.
Context	Sub-Saharan Africa (The WHO Africa Region)	Other WHO Regions such as South-East Asia, Western Pacific, Europe, and Eastern Mediterranean region
Study designs	Quantitative (observational studies), qualitative and mixed-methods study designs	Review studies such as literature review, rapid review, and expert review
Publication type	Peer reviewed papers	Abstracts only, conference documents, editorials, non-peer-reviewed publication such as reports, theses and dissertations
Language	All publication languages	
Time frame	Publications within 10 years (between 2012 and 2023)	

### Literature searches

The purpose of our search was to identify relevant peer-reviewed papers that address the review questions. To accomplish this, a comprehensive search was conducted across several electronic databases, including PubMed, EBSCOhost (CINAHL, PsycInfo, and Health Source: Nursing/Academic Edition), SCOPUS, and Web of Science for original articles published within between 2012 and 2023. Additionally, a search using the Google Scholar search engine was performed to identify additional literature of relevance. For the database searches, we developed a search strategy in collaboration with an information scientist, ensuring the inclusion of relevant keywords such as “survivor,” “gender-based violence,” “sexual violence,” “injuries,” and “trauma.” We employed Boolean operators (AND/OR) and Medical Subject Heading (MeSH) terms to refine the search string (Please refer to Supplementary File 1 for the detailed search strategy). Adjustments were made to the syntax based on the specific requirements of each database. The information scientist also played a role in conducting website searches. In addition to electronic searches, we manually explored the reference lists of included sources to identify any additional relevant literature. At this stage, no search filters based on language or publication type were applied, however, the search results will be limited to publications from 2012 to 2023. This date limitation was to enable as captured recent and relevant studies to understand the current trend. All search results were imported into an EndNote Library X20 for efficient citation management.

### Articles selection process

A study selection tool was developed using Google Forms based on the items outlined in the inclusion criteria (Table 1) and was subsequently pilot tested. The EndNote library was then examined for duplicates using the “Find Duplicate” function. Two authors (DK and ML) independently utilised the study screening tool to categorise titles and abstracts into two groups: “include” and “exclude.” Any discrepancies in their responses during this phase were resolved through discussion and consensus. The full-text articles of all titles and abstracts that met the inclusion criteria during the initial screening phase were obtained from using the Durban University of Technology Library Services, and independently screened by DK and ML following the eligibility criteria as a guide. In cases where there was a lack of consensus between DK and ML, a third author (PA) was consulted to resolve any discrepancies. The PRISMA flow diagram was utilised to document the article selection process, ensuring transparency and accountability.

### Quality appraisal

The Mixed Method Quality Appraisal Tool (MMAT) Version 2018 [37] was utilised to assess the methodological quality and potential risk of bias in the included studies. This tool was employed to evaluate the appropriateness of the study’s objective, the suitability of the study design, participant recruitment methods, data collection procedures, data analysis techniques, and the presentation of results/findings. To determine the quality of the studies, a quality score based on established criteria was applied, where a score of 50% indicated low quality, 51–75% indicated average quality, and 76–100% indicated high quality. The total percentage score was calculated by adding all the items rated, divided by seven, and multiply by a hundred. This rigorous assessment is crucial for identifying any research gaps. Two authors (DK and ML) independently conducted the quality appraisal, and any disagreements were resolved by involving a third author (PA).

### Data charting

Data extraction was conducted using a spreadsheet, which underwent a pilot test with 15% (3) of the included evidence sources to ensure its efficacy in capturing all relevant data for addressing the review question. Feedback from the pilot test was carefully considered, and necessary adjustments were made to the form. Upon a comprehensive examination of the full texts, two independent reviewers (DK and ML) extracted all pertinent data from the included studies. The data extraction process employed a hybrid approach, incorporating both inductive and deductive reasoning [38]. The process involved a thorough analysis of the extracted information to identify patterns, themes, and trends in the existing research evidence regarding injuries and trauma resulting from SGBV among survivors in sub-Saharan Africa. Key study characteristics, including author(s), publication year, study title, aim/objective, geographical location (country), study design and study population, were extracted. Additionally, the study findings pertaining to injuries and/or trauma resulting from SGBV were recorded.

### Collating, summarising, and reporting the results

The results of the data extraction were collated and summarised in a narrative format. Descriptive analysis and narrative synthesis were utilised to present the findings in a comprehensive manner. The study outcomes included a comprehensive overview of the scope of research evidence on injuries and trauma due to SGBV among survivors in the region. This study was be reported in keeping with the Preferred Reporting Items for Systematic reviews and meta-analyses extension for scoping reviews (PRISMA-ScR) checklist [39].

## Results

### Study selection

**Fig. 1:**
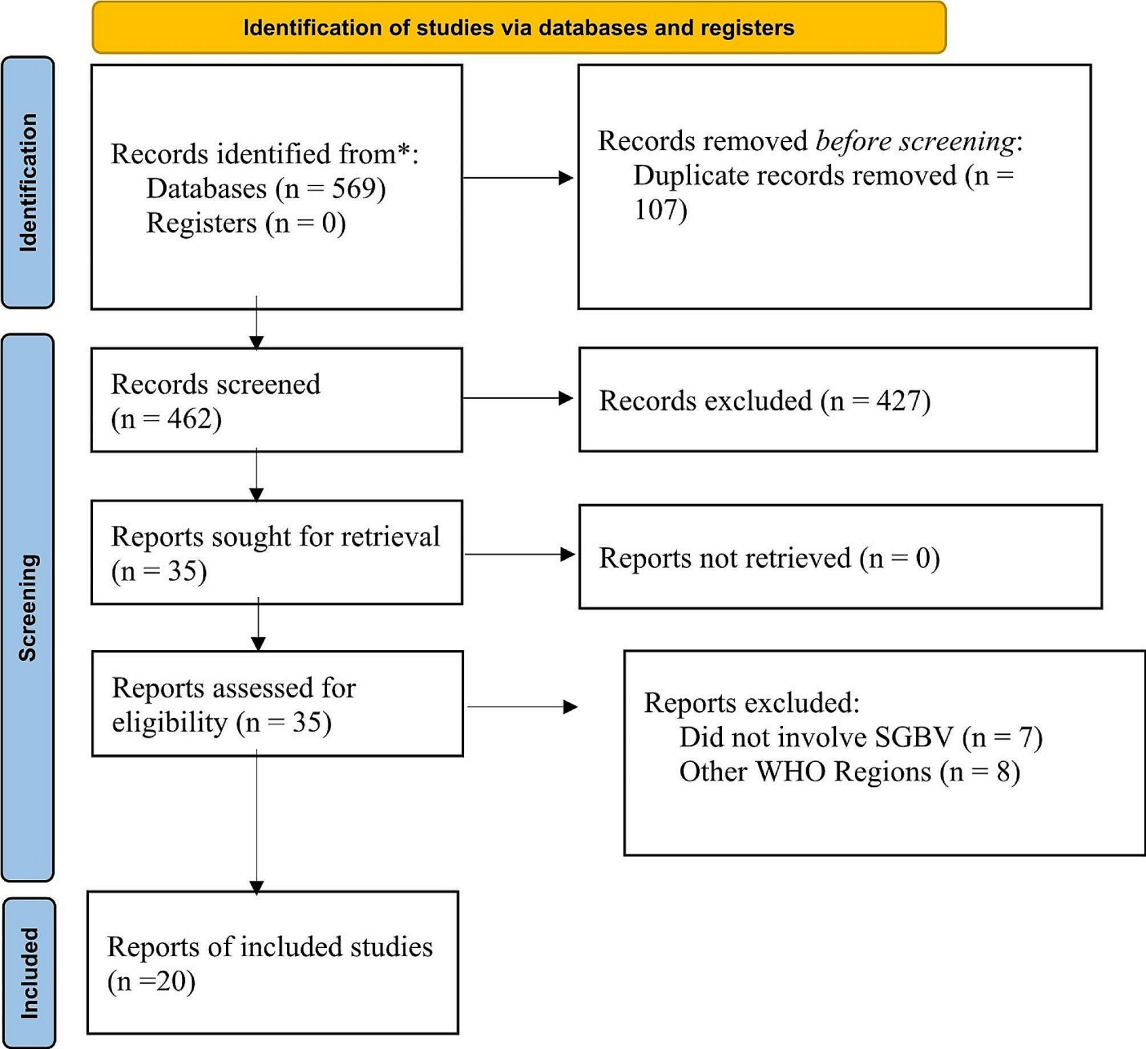
PRISMA 2020 flow diagram

A total of 569 potentially eligible titles and abstracts across databases were screened and after excluding duplicates and those that did not meet this eligibility criteria, included 20 [13–32] studies for data extraction and analysis (Fig. 1).

### Characteristics and quality appraisal of the included studies

Of the 20 included studies, the majority (*n* = 4; 20%) were from South Africa, and mostly (*n* = 11; 55%) published between 2012 and 2022. The majority (*n* = 9, 45%) were cross-sectional studies, and mostly (*n* = 15; 75%) involved women. The mean quality score ± SD of the 20 included studies was 87% ± 13. All details on the characteristics and quality appraisal of the included studies are provided in Table 2.

**Table 2:** Characteristics and quality appraisal of the included studies (*N* = 20)

Characteristics	*n* (%)
**Study country**	
South Africa [17, 24–26]	4 (20.0)
Nigeria [13, 14, 29]	3 (15.0)
Kenya [13, 14, 29]	3 (15.0)
Uganda [18, 22, 23]	3 (15.0)
Ghana [15, 16]	2 (10.0)
Ethiopia [19, 28]	2 (10.0)
Democratic Republic of Congo [30]	1 (5.0)
Togo [31]	1 (5.0)
Zimbabwe [21]	1 (5.0)
***Year of publication***	
2012–2017 [13, 17, 20, 21, 23, 25, 26, 30, 31]	9 (45.0)
2018–2022 [14–16, 18, 19, 22, 24, 27–29, 32]	11 (55.0)
**Study design**	
Cross-sectional study [13, 17, 19, 20, 23, 25, 28, 31, 32]	9 (45.0)
Qualitative study [14–16, 22]	4 (20.0)
Cohort study [18, 24, 26, 29]	4 (20.0)
Mixed-methods study [21, 30]	2 (10.0)
Case series [27]	1 (5.0)
**Study population**	
Sexually abused children [13]	1 (5.0)
Married women [15]	1 (5.0)
Visually impaired women [16]	1 (5.0)
Male and females refugee asylum seekers survivors of SGBV [22, 23]	2 (10.0)
Female student (high school, undergraduate, and private college) [19, 20, 28]	3 (15.0)
Adolescents [14]	1 (5.0)
Female sex workers [29]	1 (5.0)
Women in reproductive age group [21]	1 (5.0)
Males and females [18]	1 (5.0)
HIV-uninfected and HIV-infected women [31]	1 (5.0)
Women (unspecified) [17, 24–27, 30, 32]	7 (35.0)
**Quality appraisal score**	
Between 50 and 75% [13–17, 21, 22, 27]	8 (40.0)
75% and above [18–20, 23–26, 28, 29, 31, 32]	12 (60.0)

### Study findings

#### Theme 1: physical injuries/trauma due to SGBV occurrence/prevalence, pattern, and associated factors

Several studies have explored the prevalence and factors associated with injuries/trauma due to SGBV (Table 3). Ssewanyana et al. highlighted the occurrence of genital trauma among adolescent girls resulting from sexual assault [14]. Apatinga et al. demonstrated that sexual violence was accompanied by physical abuse, leading to physical injuries among women [15]. Azumah et al. reported that visually impaired women who experienced gender-based violence faced a higher risk of injuries including genital injuries [16]. Amashnee et al. identified specific patterns in the occurrence of sexual assault injuries, with higher prevalence on Mondays (28%) and Fridays (27.3%), during specific months, and predominantly during working hours [17]. Abubeker et al. examined the impact of physical violence on female students, with findings indicating various injuries such as bruising, cuts, scratches, and fractures, leading to missed classes and fear of walking alone [19]. Biribawa et al. investigated the burden of GBV-related injuries and found a significant number of hospital visits in Uganda, with slightly declining injury rates (from 13.6 to 13.5 per 10,000 population) from 2012 to 2016 [18]. Umana et al. documented that 6.6% of undergraduate and postgraduate female students experienced sexual intimate partner violence, leading to injuries such as cuts, bruises, and sprains [20]. Mukanangana et al. reported the prevalence of virginal bleeding, genital irritation and urinary tract infection among women in reproductive age in Zimbabwe [21]. These findings collectively underscore the occurrence/prevalence physical injuries/trauma, pattern and specific associated factors associated resulting from SGBV.

**Table 3:** Physical injuries/trauma due to SGBV occurrence/prevalence, pattern, and associated factors

Author, year, and country	Study aim/objective	Key findings/results
Ssewanyana et al. 2019 [14] Kenya	To explore views from young people and local stakeholders on forms and underlying factors for unintentional injury, violence, self-harm, and suicidal behaviour of adolescents in Kilifi County.	• Genital trauma resulting from sexual assault was experienced by many adolescent girls.
Apatinga et al., 2021 [15] Ghana	To explore the consequences of sexual violence against married women in the Eastern Region	• Following sexual violence and abuse, women experienced physical injuries.
Azumah et al., 2019 [16] Ghana	To find answers to GBV against visually impaired persons in the Kumasi and their experiences in relation to GBV	• GBV had effects of battering against visually impaired women, leading to a greater risk of injuries such as genital injuries.
Amashnee et al., 2016 [17] South Africa	To describe the trend and profile of interpersonal violence-related injuries in an emergency department and crisis centre at the Leratong Provincial Hospital.	• Of the 107 females, sexual assault injuries presented mainly on Mondays (*n* = 30, 28%) and Fridays (*n* = 27, 27.3%). • Victims of sexual assault presented predominantly during December (*n* = 12; 15.8%), March (*n* = 12; 21.1%), January (*n* = 10; 14.5%), February (*n* = 10; 23.8%) and October (*n* = 10; 16.4%) • Victims of sexual assault most often presented at 11h00 • Sexual assault cases showed an increase during normal working hours 08h00 to 16h00 (*n* = 82; 32%), with a decline after 16h00 (*n* = 24) • Sexual assault was the most common type of injury in the age group 10–19 years (*n* = 63; 47.4%)
Biribawa et al., 2022 [18] Uganda	To describe the trends and distribution of injuries due to GBV	• GBV resulted in 49,842 (0.12%) of hospital (outpatient) visits in Uganda in 2016. • Injury rates due to GBV were 13.5 per 10,000 population for the year 2012, and 13.6 per 10,000 population for 2016. • Injury rates declined slightly from 2012 to 2016 (OR: 0.995, 95% CI: 0.991–0.997). • In 2016, the odds of injuries due to GBV were 1.36 times in females compared to males (OR: 1.36, 95% CI: 1.33–1.38).
Abubeker et al. 2021 [19] Ethiopia	To assess the prevalence and associated factors of GBV among 2nd and 3rd year female students at private colleges in Harar town	• Of the 108 female students, 34 (31.5%) of the survivors experienced multiple acts of physical violence. • Bruising (*n* = 60, 55.6%) was the most common injury committed to them, followed by cuts (*n* = 28, 25.9%), scratches (*n* = 14, 12.9%), and fractures (*n* = 6, 5.6%). • 62 (57.4%) of the survivors had missed classes due to the condition, 2 (1.9%) experienced fear to go to the library in dark, and 17 (15.7%) feared to walk alone.
Umana et al. 2014 [20] Nigeria	To assess the prevalence and correlates of IPV in female undergraduate and postgraduate students in a tertiary institution.	• Out of 1,100 undergraduate and 255 postgraduate female students, the proportion who had experienced sexual intimate partner violence was 6.6%. • Victims sustained injuries such as bites, scratches, abrasions, bruises; and sprains, and dislocations
Mukanangana et al. 2014 [21] Zimbabwe	To identify the forms of GBV and their reproductive health impacts to women in the reproductive age in specifically in Hatcliffe area.	• Out of the 126 women in the reproductive age group (15–49 years), 95% experienced physical violence, 31% reported rape by a stranger, and 92% reported spousal rape. • Vaginal bleeding, genital irritation and urinary tract infection were also reported by 78%, 76% and 72% of respondents respectively in the multiple responses question.

#### Theme 2: consequences and impact on mental health

Several studies highlighted the significant consequences and impact of SGBV on mental health (Table 4). Ombok et al. found that sexually abused children had a high prevalence (49%) of post-traumatic stress disorder (PTSD), which was associated with the duration of abuse, severity of injuries sustained, parents’ marital status, and family dynamics [13]. Apatinga et al. demonstrated that sexual violence was accompanied by emotional abuse, leading to psychological problems, sexual and reproductive health issues, and suicidal ideations among women [15]. Azumah et al. reported that visually impaired women who experienced gender-based violence faced a higher risk of suicide attempts, and marital breakdown [16]. Liebling et al. found that women and girls who experienced SGBV frequently became pregnant and suffered from injuries, disability, and psychological trauma [22]. Morof et al. highlighted the high prevalence of violence and its association with PTSD symptoms and depression among women [23]. Nguyen et al. demonstrated that exposure to various forms of gender-based violence, including intimate partner violence and sexual harassment, was significantly associated with hypertension, mediated by depression, post-traumatic stress symptoms, and alcohol binge-drinking [24]. Abrahams et al. reported that women raped by intimate partners had higher levels of depressive symptoms compared to those raped by strangers [25]. Pitpitan et al. found a significant association between gender-based violence and increased alcohol use, as well as heightened levels of depressive symptoms and PTSD symptoms [26]. Okunola et al. revealed the complications experienced by survivors of sexual assault, including sexually transmitted infections, depression, and post-traumatic stress disorder [27]. Umana et al. identified the negative impact of violence on academic performance, with victims experiencing loss of concentration, self-confidence, and school absenteeism [20]. Roberts et al. highlighted the association between severe GBV and higher depressive symptoms, PTSD symptoms, disordered alcohol use, and more sex partners [29]. Tantu et al. emphasized the wide range of social, health-related, and psychological consequences resulting from gender-based violence [28]. Finally, Mukanangana et al. revealed that the majority of respondents who experienced rape suffered from psychological trauma, exposure to sexually transmitted infections, unwanted pregnancies, loss of libido, and illegal abortions [21]. These findings collectively demonstrate the significant impact of SGBV on mental health, including psychological trauma, depression, PTSD symptoms, and various adverse outcomes.

**Table 4:** Consequences and impact on mental health

Author, year, and Country	Form of gender-based violence reported	Key finding/results
Ombok et al., 2013 [13] Kenya	Sexual abuse	• The prevalence of PTSD among sexually abused children was 49%. • PTSD was significantly associated with shorter duration of sexual abuse, greater severity of injuries sustained during assault, parents’ marital status, and the family’s way of sorting out disagreements.
Apatinga et al., 2021 [15] Ghana	Sexual violence	• Following sexual violence and abuse, women experienced psychological problems, sexual and reproductive health problems, and suicidal ideations. • These health difficulties significantly undermined their economic activities and depleted their income.
Azumah et al., 2019 [16] Ghana	GBV (unspecified)	• GBV had effects of battering against visually impaired women, leading to a greater risk of suicide attempts, lack of love and trust in marriages, marital breakdown, unwanted pregnancy, and abortion.
Liebling et al. 2020 [22] Uganda	Rape	• Women and girls frequently became pregnant following rape on their journey to settlements. • Male and female refugees often couldn’t work due to injuries, including genital damage, disability due to torture, and psychological trauma.
Morof et al. 2014 [23] Uganda	Physical violence and sexual violence	• Of the 500 women, lifetime prevalence of any violence, physical violence, and sexual violence was 77.5%. • Physical and sexual violence were associated with PTSD symptoms and depression.
Nguyen et al. 2022 [24] South Africa	Intimate partner violence, non-partner sexual violence, and sexual harassment	• Exposure to GBV (intimate partner violence and sexual harassment) were significantly associated with hypertension in young women, mediated by depression, post-traumatic stress symptoms, and alcohol binge-drinking.
Abrahams et al. 2013 [25] South Africa	Rape	• Women raped by intimate partners had higher levels 84.3% of depressive symptoms compared to those raped by strangers.
Pitpitan et al. 2012 [26] South Africa	GBV (unspecified)	• Increased alcohol use, as well as heightened levels of depressive symptoms and PTSD symptoms were significant associated with GBV.
Okunola et al. 2021 [27] Nigeria	Sexual assault	• Of the 74 survivors of sexual assault 13.5% experienced complications such as sexually transmitted infections, depression (4.1%), and PTSD (5.4%).
Umana et al. 2014 [20] Nigeria	Sexual intimate partner violence	• Of the 1355 respondents, the academic performance of 71.1% victims was affected as violence caused loss of concentration loss of self-confidence (68.9%), and school absenteeism (56.0%).
Tantu et al. 2020 [28] Ethiopia	GBV (unspecified)	• GBV resulted in social problems, health-related problems (e.g., unwanted pregnancy, abortions, vaginal discharge, and injury to genitals), and psychological complications (e.g., self-blame, anxiety, suicidal attempts).
Roberts et al. 2018 [29] Kenya	GBV (unspecified)	• Severe GBV was associated with higher depressive symptoms, PTSD symptoms, disordered alcohol use, and more sex partners. • Sexual GBV was associated with disordered alcohol use. • Physical/Moderate Emotional GBV was associated with more sexual partners and a higher prevalence of unprotected sex.
Mukanangana et al. 2014 [21] Zimbabwe	Rape	• Of 126, most respondents, 89%, 88%, 83%, 82% and 80% in a multiple response question suffered psychological trauma (89%), exposure to sexually transmitted infections (including HIV) (88%), unwanted pregnancies (83%), loss of libido (82%) and illegal abortions (80%) because of rape.

#### Theme 3: healthcare access and support services

The findings from the studies conducted in the Democratic Republic of the Congo and Togo highlight significant barriers and challenges faced by survivors of SGBV in accessing healthcare and receiving proper psychological care. In the Democratic Republic of the Congo, Scott et al. reported that SGBV survivors faced barriers to accessing healthcare, such as availability and affordability, in their study to evaluate community attitudes of SGBV and health facility capacity to address SGBV in the eastern part of the country [30]. Access to mental health care was difficult [30]. Witch doctors and other traditional healers provided mental health services to some survivors [30]. Burgos-Soto et al.‘s study in Togo, which sought to estimate the prevalence and contributing factors of intimate partner physical and sexual violence among HIV-infected and -uninfected women, found that lifetime prevalence rates of physical and sexual violence were significantly higher among HIV-infected women compared to uninfected women [31]. 42% of the women admitted to ever suffering physical harm as a result of intimate partner abuse [31]. Only one-third of the injured women had ever told the medical professionals the true nature of their injuries, and none had been directed to neighbourhood organizations for the proper psychological care [31].

#### Theme 4: rural vs. urban disparities

According to a study conducted in Nigeria by Na et al. to identify the trends in sexual assault against women in urban and rural areas of Osun State, completed rapes occurred 10.0% of the time in urban areas and 9.2% of the time in rural areas, while attempted rapes occurred 31.4% of the time in urban areas and 20.0% of the time in rural areas [32]. Rural women were more likely than urban women to endure repeated sexual assault and non-genital injuries [32]. This study findings suggest that sexual assault against women occurs in both urban and rural areas, with notable differences in the patterns and outcomes.

## Discussion

This scoping review study on injuries and trauma resulting from sexual and SGBV) in sub-Saharan Africa revealed key findings that shed light on this critical issue. The majority (15%) of the included studies were conducted in South Africa. Most (75%) of these studies adopted a cross-sectional design and focused on women as the population of interest. The overall mean quality score of the included studies was high, indicating robustness and reliability in the research.

The findings from the included studies collectively highlighted the prevalence of physical injuries and trauma resulting from SGBV in sub-Saharan Africa such as genital injuries, cuts, bites, scratches, abrasions, bruises, sprains, dislocations, fractures, vaginal bleeding, and genital trauma. The included studies provided insights into the consequences and specific factors associated with such violence, emphasising the urgent need for effective interventions and support services. Notably, the impact of SGBV on mental health was a recurring theme in the literature, with evidence pointing to psychological trauma, depression, PTSD symptoms, and other adverse outcomes experienced by survivors.

While the review identified limited research on healthcare access and support services for SGBV survivors, the available studies underscored significant barriers in accessing healthcare and receiving proper psychological care [30, 31]. Challenges included limited availability and affordability of services, as well as survivors’ hesitancy to disclose abuse to medical professionals. These findings highlight importance healthcare gaps requiring interventions to ensure comprehensive support for survivors in sub-Saharan Africa.

Policymakers in sub-Saharan Africa should prioritise the implementation of comprehensive and evidence-based interventions to address injuries and trauma resulting from SGBV. The concentration of included studies from South Africa indicates the need to expand research efforts to include other countries in the region, ensuring that policies are tailored to meet the diverse needs and contexts of different nations. The limited research on healthcare access and support services for SGBV survivors underscores the urgency of improving healthcare systems and strengthening support services for survivors. Policymakers should consider investing in accessible and affordable healthcare services that provide specialised care for SGBV survivors, including mental health support. Additionally, addressing publication language bias by promoting research in multiple languages (e.g., French and Portuguese) can ensure that relevant findings reach policymakers across the sub-Saharan African region. Furthermore, this scoping review’s potentially can inform the development of targeted policies that address the specific risk factors, consequences, and contributing factors associated with injuries and trauma resulting from SGBV.

The scoping review findings highlight several avenues for future research on injuries and trauma as a result of SGBV in sub-Saharan Africa. Researchers should focus on conducting studies in countries with limited representation in the current literature to enhance the breadth and diversity of evidence available. Investigating the barriers and challenges faced by survivors in accessing healthcare and support services should be a priority to identify gaps and improve service delivery. Moreover, longitudinal studies could provide valuable insights into the long-term consequences of SGBV on survivors’ mental health and well-being. Researchers should also explore the effectiveness of various interventions, including those involving community-based support systems, to address SGBV-related injuries and trauma. Furthermore, incorporating qualitative research approaches could deepen the understanding of survivors’ experiences and help in tailoring interventions to their specific needs. Future research should also consider the perspectives of various stakeholders, including healthcare providers, community leaders, and policymakers, to develop comprehensive and context-specific strategies to prevent and respond to SGBV and its consequences. Overall, conducting rigorous research that spans diverse contexts and populations will contribute to a more comprehensive understanding of the multifaceted challenges posed by SGBV and inform evidence-based interventions that promote survivor support and well-being.

The scoping review’s strength lies in its comprehensive approach, encompassing a wide range of literature on injuries and trauma resulting from SGBV in sub-Saharan Africa. By considering various study designs and sources of evidence, the review offers a holistic view of the topic. Additionally, the study effectively identifies key themes and trends in the literature, leading to a deeper understanding of the prevalence, consequences, and specific factors associated with injuries and trauma resulting from SGBV in the region. The mapping of research evidence within the review proves to be a valuable resource for researchers, policymakers, and practitioners working in the field of SGBV. Furthermore, the review’s emphasis on studies with an overall high mean quality score (87% ± 13%) enhances the credibility and reliability of the findings, ensuring that the evidence presented is robust and trustworthy.

Despite these strengths, this scoping review has several limitations. The concentration of included studies from South Africa introduces a geographic bias, potentially limiting the generalizability of findings to other countries within sub-Saharan Africa. To enhance the review’s applicability, a more diverse representation of research from different regions in the area would be beneficial. Additionally, the paucity of studies investigating healthcare access and support services for SGBV survivors may restrict the review’s ability to provide comprehensive insights into this critical aspect of the topic. Despite these limitations, this scoping review provides a valuable overview of the available research evidence on injuries and trauma related to SGBV in sub-Saharan Africa, paving the way for further research and targeted interventions to address this critical issue. Researchers should acknowledge and consider these limitations when interpreting and applying the review’s findings.

In conclusion, this scoping review provides a comprehensive overview of the research evidence on injuries/trauma resulting from SGBV in the sub-Saharan African region. It underscores the urgent need for further research and targeted interventions to address this pervasive issue and support the well-being of survivors.

### Electronic supplementary material

Below is the link to the electronic supplementary material.

Supplementary Material 1

## Data Availability

All data sources will be presented in a form of references.
