# Supplementary material for: Injuries and /or trauma due to sexual gender-based violence among survivors in sub-Saharan Africa: a systematic scoping review of research evidence
Source: Arch Public Health. 2024 May 21;82:78. doi: 10.1186/s13690-024-01307-3 (PMC11106996; doi:10.1186/s13690-024-01307-3)
Supplement: Supplementary file 1 — Supplementary Material 1 [file 13690_2024_1307_MOESM1_ESM.docx]

**Supplementary file:** Literature searches

| Date | Database | Search string /keywords | Search yield | Number potentially eligible |
| --- | --- | --- | --- | --- |
| 01/07/2023 | PubMed | **#1:** Search: injury OR trauma  "injurie"[All Fields] OR "injuried"[All Fields] OR "injuries"[MeSH Subheading] OR "injuries"[All Fields] OR "wounds and injuries"[MeSH Terms] OR ("wounds"[All Fields] AND "injuries"[All Fields]) OR "wounds and injuries"[All Fields] OR "injurious"[All Fields] OR "injury s"[All Fields] OR "injuryed"[All Fields] OR "injurys"[All Fields] OR "injury"[All Fields] OR ("injuries"[MeSH Subheading] OR "injuries"[All Fields] OR "trauma"[All Fields] OR "wounds and injuries"[MeSH Terms] OR ("wounds"[All Fields] AND "injuries"[All Fields]) OR "wounds and injuries"[All Fields] OR "trauma s"[All Fields] OR "traumas"[All Fields])  **#2:** Search: sexual violence OR gender-based violence OR sexual assault  "sex offenses"[MeSH Terms] OR ("sex"[All Fields] AND "offenses"[All Fields]) OR "sex offenses"[All Fields] OR ("sexual"[All Fields] AND "violence"[All Fields]) OR "sexual violence"[All Fields] OR ("gender based violence"[MeSH Terms] OR ("gender based"[All Fields] AND "violence"[All Fields]) OR "gender based violence"[All Fields] OR ("gender"[All Fields] AND "based"[All Fields] AND "violence"[All Fields]) OR "gender based violence"[All Fields]) OR (("sexual behavior"[MeSH Terms] OR ("sexual"[All Fields] AND "behavior"[All Fields]) OR "sexual behavior"[All Fields] OR "sexual"[All Fields] OR "sexually"[All Fields] OR "sexualities"[All Fields] OR "sexuality"[MeSH Terms] OR "sexuality"[All Fields] OR "sexualization"[All Fields] OR "sexualize"[All Fields] OR "sexualized"[All Fields] OR "sexualizing"[All Fields] OR "sexuals"[All Fields]) AND "assult"[All Fields])  **#3:** Search: (injury OR trauma) AND (sexual violence OR gender-based violence OR sexual assault)  **#4:** Search: (injury OR trauma) AND (sexual violence OR gender-based violence OR sexual assault) Filters: in the last 10 years | 1,940,086  43,439  8,826  4,816 | 271 |
| 02/07/2023 | EBSCOhost (Academic Search Complete, PsycInfo, and Health Source: Nursing/Academic Edition) | <https://web-s-ebscohost-com.ukzn.idm.oclc.org/ehost/breadbox/search?term=SU%20Injury%20OR%20injuries%20OR%20trauma%20AND%20sexual%20violence%20OR%20gender-based%20violence%20AND%20women%20AND%20African%20countries&sid=9b9d1881-8a50-4819-8016-05d64645ebc8%40redis&vid=27> | 1,058 | 92 |
| 04/07/2023 | SCOPUS | TITLE-ABS-KEY (injury OR injuries OR trauma AND sexual AND violence OR gender-based AND violence AND women AND africa AND countries) | 34 | 4 |
| 04/07/2023 | Web of Science | Injury OR injuries OR trauma AND sexual violence OR gender-based violence AND women AND African countries | 2,326 | 102 |
| 10/07/2023 | Google Scholar | Injury OR injuries OR trauma AND sexual violence OR gender-based violence AND women AND African countries | First 500 of 16,800 | 100 |
